# ‘Nature Concocts & Expels’: The Agents and Processes of Recovery from Disease in Early Modern England

**DOI:** 10.1093/shm/hkv022

**Published:** 2015-03-26

**Authors:** Hannah Newton

**Keywords:** recovery, concoction, expulsion, Nature, Helmontians

## Abstract

The ‘golden saying’ in early modern medicine was ‘Nature is the healer of disease’. This article uncovers the meaning and significance of this forgotten axiom by investigating perceptions of the agents and physiological processes of recovery from illness in England, *c*.1580–1720. Drawing on sources such as medical texts and diaries, it shows that doctors and laypeople attributed recovery to three agents—God, Nature and the practitioner. While scholars are familiar with the roles of providence and medicine, the vital agency of Nature has been overlooked. In theory, the agents operated in a hierarchy: Nature was ‘God's instrument’, and the physician, ‘Nature's servant’; but in practice the power balance was more ambivalent. Nature was depicted both as a housewife who cooked and cleaned the humours, and as a warrior who defeated the disease. Through exploring these complex dynamics, the article sheds fresh light on concepts of gender, disease and bodies.

## Introduction

In November 1675, the Essex vicar Anthony Walker ‘grew very ill’ from pleurisy. His wife, Elizabeth, reported, he ‘groan'd all Night’, with ‘tremblings, and a fumbling in his Speech[,] [which] bad Symptoms gave me fear of the sudden approach of Death’. Sending for doctors from London, he was let blood twice, but to no avail. After the second bleeding, Anthony ‘stretched out [his] left Arm’, and demanded, ‘I would Bleed again’. He explained, ‘I … bled at [the] Nose, [and] Nature indicated thereby what must relieve’. The physicians, initially reluctant to repeat the procedure, consented, and to Anthony's great relief, ‘Blood sprang out so abundantly, that they drew at least ten Ounces’. He concluded, ‘my last … Bleeding … saved my life, without which … I could not have escaped; blessed be God, who put that Resolution into my Mind’.^1^

This account is taken from the autobiography of Anthony's wife, Elizabeth, published in 1694. It raises questions about early modern understandings of recovery from illness. Anthony implies that three parties had played a role: God, Nature and physicians. How did these agents fit together and interact? What, exactly, did Anthony mean by ‘Nature indicated thereby what must relieve’? Taking the perspectives of doctors and laypeople, this article investigates the agents and physiological processes of recovery from illness in England between approximately 1580 and 1720.

There is a rich historiography on early modern theories of disease and treatment. Scholars have shown that illness was attributed to the malignant alteration of the body's ‘humours’, the four special fluids that made up the body, and medicine was designed to expel or correct these humours.^2^ Treatments included oral and topical remedies, surgical procedures, and the regulation of the ‘non-naturals’, the six environmental and dietary factors that were thought to affect the body—air, diet, exercise, sleep, evacuation and the passions.^3^ Such insights are valuable, but they do not constitute a comprehensive picture of early modern explanations of recovery. By concentrating on medical intervention, other agents and mechanisms that were thought to play a role have been disregarded. This article seeks to expand our knowledge of early modern perceptions of recovery by looking beyond medical treatment. In particular, I draw attention to the vital agent mentioned by Anthony Walker, Nature. Nature is ubiquitous in accounts of illness and recovery, and yet with a few notable exceptions discussed below, the therapeutic role of this agent has been overlooked by historians.

The reason for this comparative neglect may be that the word ‘nature’ is so common in today's parlance that we barely notice it when it appears in early modern texts. If we do pause to consider the meaning of this word, we usually assume it refers to the broader concept of the physical world, or to some spontaneous ‘natural’ process. In this article, I show that such a reading is mistaken: in the early modern period, ‘Nature’ denoted a specific bodily agent which acted intelligently to restore health. Personified as a benevolent woman who inhabited the body, Nature proved to be a resilient and enduring concept, widely recognised throughout the early modern period. These findings have important implications for the history of medicine: namely, the whole rationale behind medical treatment, together with understandings of how the body worked, rested on the precept that Nature is the healer of disease. The article also examines the complex gender and power dynamics between female Nature and the male physician, and suggests that these interactions offer fresh insights into wider cultural attitudes to womankind. Historians have explored doctors' relationships with their female patients, and the gendering of human bodies, but the female personification of Nature in the body has not been considered.^4^

The most extensive historical study of Nature's role in recovery is a monograph written by the German scholar Max Neuburger in 1926.^5^ Neuburger covered a vast swathe of history, from ancient times to the present day, but he limited his enquiry to the views of learned physicians. By focusing on a shorter period, I seek to provide a more nuanced account, which encompasses the opinions of laypeople as well as doctors. More recently, Gianna Pomata has investigated the phenomenon of ‘male menstruation’, which was interpreted as ‘the healing endeavour of nature herself’.^6^ Building on Pomata's findings, this article investigates a greater range of mechanisms through which Nature removed disease. The agency of Nature has also featured in case studies of particular physicians. Barbara Duden's analysis of the medical practice of the eighteenth-century German doctor, Johann Storch, discusses the ‘efforts on the part of nature … to restore the body to good health’.^7^ In an English context, Andrew Wear and Andrew Cunningham have evaluated the theories of the seventeenth-century physician Thomas Sydenham in relation to Nature's role, suggesting that his emphasis on this agent was especially pronounced.^8^ Here, I explore the views of a larger assortment of individuals, demonstrating that the belief in Nature's healing powers was widespread.

Since this study is about the agents and processes of recovery, it is necessary to define this term. Recovery denoted the ‘translation of the *disease* into *health*’, and it comprised two stages: ‘the away-taking of the *Disease*’, followed by the restoration of strength (or convalescence).^9^ This article is about the first part. Convalescence deserves a separate study.^10^ Various words were used interchangeably with ‘recover’, of which the most common were ‘cure’, ‘heal’ and ‘deliver’.^11^ Of these terms, ‘recover’ has been privileged in this article on the grounds that it is the most neutral, and does not carry the strong medical or religious connotations which are associated with some of the other words.

To set the parameters of this investigation, it concentrates on recovery from acute physical illness. It is hoped that this initial research will lead to comparative studies with other conditions, such as chronic disease, childbirth, surgery and mental illness. Due to pressure of space, the article will not consider the roles of magic or astrology in recovery, nor will it include those cases where God was believed to have intervened directly, rather than through secondary causes. Finally, the patient's emotional and spiritual experience of recovery is not considered here; this subject is addressed at length elsewhere.^12^

This study draws on a range of sources. To access medical opinions, vernacular medical texts of various genres have been used, including general ‘methods of physic’, texts which describe the fundamental principles behind medicine, such as the role of the physician in relation to the other agents of recovery.^13^ Treatises about particular diseases provide more detailed accounts of the effects of remedies, through which it is possible to piece together what was thought to be happening inside the body during recovery. Books about Nature, a miscellaneous group of medical and philosophical treatises, describe explicitly Nature's role in recovery. Examples include *The Secret Miracles of Nature*, by the Dutch physician Levinus Lemnius (1505–68), and a critical exposition of mainstream views of Nature by the natural philosopher and chemist Robert Boyle (1627–91).^14^ These texts are supplemented by a number of doctors' casebooks, documents which purport to describe the treatments of real patients. The notebook of the eminent Stratford puritan physician, John Hall (1575–1635), for example, contains biographical information about 125 of the 178 patients, from which it is possible to authenticate the histories.^15^ Regardless of whether the outcomes of the cases were genuine, they reveal how contemporaries explained recovery.

The above medical texts were all published in early modern England, and most of the authors claimed to be physicians or surgeons from Britain or Europe. Nevertheless, it is not always easy to date the information contained within the texts, nor to appropriate it to particular individuals. This is due to the tendency of writers to plagiarise one another, copying and pasting ideas, and sometimes whole paragraphs, from ancient, medieval and contemporary works.^16^ Furthermore, many of the texts are translations of earlier works, often published years after the death of the original author. The surgical treatise of the distinguished French military surgeon, Ambroise Paré (*c*.1510–90), for example, was not published in England until 1634, 44 years after Paré's death; it had first appeared in Antwerp in 1579, and was then translated into English by one Thomas Johnson.^17^ Inevitably, editors and translators altered the texts, sometimes incorporating their own thoughts along the way. In view of these issues, the ideas about recovery conveyed within the texts must be regarded as representative not so much of the individual authors, but of a patchwork of viewpoints from before and beyond the lifespans of those physicians named on the title pages.

The intended audiences of the above medical texts were wide. *Praxis medicinae, or, the physicians practice* (1632), by the German physician Walter Bruele, was ‘published for the good, not onely of Physicians, Chirurgions, and Apothecaries, but very meete and profitable for all such which are solicitious of their health’.^18^ Laypeople's demand for this sort of information is indicated by the fact that many of the texts went through multiple editions. Nevertheless, even if people were reading these texts, we cannot know for certain whether they agreed with the books' contents. Thus, in order to discover more about lay beliefs concerning recovery, it is necessary to use sources penned by laypeople themselves, such as diaries and letters. Although these sources underrepresent the lower socioeconomic groups, it is occasionally possible to infer poorer people's ideas from second-hand reports about servants and other individuals.

The majority of the sources cited in this research draw on the Hippocratic–Galenic tradition, by which I mean they subscribe to the humoral theory of disease and treatment.^19^ However, in recognition that this type of medicine did face a degree of opposition in the period, a section of the article is devoted to the beliefs of the Helmontians, followers of the Flemish physician and chemist Jan Baptista van Helmont (1579–1644).^20^ The purpose of this case study is to demonstrate just how deeply ingrained was the role of Nature in the early modern imagination. Despite rejecting many of the fundamental tenets of Galenism, Helmontians retained the precept that Nature is the healer of disease. The comparison also suggests some new reasons for why ultimately Helmontian medicine failed to break the hegemony of Galenism, despite its promise to provide pleasant and effective remedies. The article comprises three parts: the first part identifies the agents of recovery, and explores their inter-relationships; the second section investigates the processes though which illness was overcome. The final part is a case study of the Helmontian theory of recovery.

## Agents

There were three agents of recovery, which formed a hierarchy. The first was God. In early modern English culture, the Christian God was supreme, overseeing all things, including sickness and recovery: ‘The Lord killeth, and maketh alive: he bringeth down to the grave, and bringeth up’, preached the London Presbyterian minister Timothy Rogers (1658–1728).^21^ God sent sickness as a punishment for sin, and revoked it when the patient had prayed and repented.^22^ These beliefs persisted throughout the early modern period. Little evidence has been found to support Ian Mortimer's assertion that ‘After 1690 … the religious framework to … cure had ceased to dominate attitudes to treatment’.^23^

God's instrument for removing disease was ‘Nature’, the second agent of recovery. Like today, the word ‘nature’ held many meanings, but in the context of physiology it denoted a divinely endowed power in the body.^24^ Since the body was conceived as a microcosm of the world, the Nature in the body was seen as a miniature version of the wider Nature that maintained the order of the universe.^25^ The early eighteenth-century physician and Fellow of the Royal Society, Conrade Joachim Sprengell, provided a typical definition: ‘*by the word Nature, we are to understand an Intrinsick Agent, by which the Vital motions …* absolutely necessary … to the Preservation and Restoration of human Bodies, are directed’.^26^ Nature was responsible for carrying out all the basic functions of the body, including nutrition, growth, reproduction, and most importantly here, recovery. Galen's famous text, *The Natural Faculties* confirms, ‘Nature … nourishes the animal, makes it grow, and expels its diseases … she skilfully moulds everything during the stage of genesis; and she also provides for the creatures after birth.’^27^ Without this agent, ‘there is not a single animal which could live … for the shortest time’, he concluded.^28^ Nature's vehicles for performing these functions were the ‘natural spirits’, highly rarefied, ‘subtile and Arey’ vapours, ‘raised from the purer blood’, and carried around the body in the veins.^29^ So strong was the connection between the spirits and Nature, the two were often regarded as synonymous*.*^30^ In turn, the spirits were ‘nourished’ by what was known as ‘radical moisture’ (an oily substance), and ‘innate heat’ (a glowing warmth).^31^ These were the substances in which ‘life consisteth’, which gradually depleted with age.^32^

As stated above, recovery was one of Nature's chief duties. Boyle's treatise of 1686 states, ‘Men are wont to believe, that there resides, in the Body of a sick Person, a certain Provident or Watchful Being, that … industriously employs itself … to … restore the distemper'd Body to its Pristine state of Health’.^33^ This notion was rooted in the writings of Hippocrates, and his famous axiom, ‘Natura est morborum medicatrix’, or ‘Nature is the healer of disease’.^34^ Historians usually associate this idea with the ‘New Hippocrates’, Thomas Sydenham (1624–89), but it is evident in this research that it was, in fact, articulated across the entire period.^35^

The healing role of Nature was endorsed by laypeople as well as elite physicians. The Leicestershire chaplain George Davenport (*c.*1631–77) recorded in 1655 that his friend Mr Gayer ‘began to be sick … last week … but nature stept in & relieved him’.^36^ A few decades later, the Cambridgeshire clergyman Isaac Archer (1641–1700) wrote in his diary that his wife's ‘fitts went away, and nature did it's office’.^37^ It is significant that clerics as well as physicians believed in Nature's healing role. One might have expected the former to have omitted this agent from their accounts of recovery, on the grounds that it detracted from the agency of the Lord. This does not seem to have been the case, however: the devout understood that Nature was ‘God's immediate Commissioner’, and therefore to attribute recovery to this agent did not negate the overarching role of providence.

What was Nature like? An analysis of the personification of Nature introduces an important theme that runs through the rest of this article, gender. It also provides insights into early modern perceptions of the body, and helps to explain the need for the third agent of recovery, the practitioner. Nature was personified as a benevolent female who inhabited the body. The Northampton puritan physician James Hart (d.1639), stated, ‘nature is … like a kinde and loving mother, being very solicitous and carefull of the life of man’.^38^ She was also depicted as a charwoman, who ‘scoured away’ illness, ‘sweeping every corner, [and] making the whole Body polite and trim’.^39^ Nature's economic status was lowly: the astrologer-physician Nicholas Culpeper (1616–54), called her ‘a plain homely woman in a beggarly comtemptible condition’ whose ‘wayes are very plaine[;] you may finde them in the darkest night without a Candle’.^40^ In these descriptions, the body was envisaged as a house, and disease as dirt.^41^ It made sense to depict Nature as female, because the majority of her roles fell into the category of women's work—as well as tending the sick, she was responsible for nourishment and reproduction.^42^ Carolyn Merchant has shown that the female identity of Nature was ‘age-old’, dating back to pagan times; in medieval and early modern Europe, feminine nouns were used to denote this agent.^43^

The female personification was not as simple as it seems, however: whilst continuing to use feminine pronouns, authors deployed masculine metaphors. ‘Dame Nature’, declared Culpeper, ‘iss like a Prince in the body … she can expell her enemy out of her dominions’.^44^ She was ‘a wise and faithful consul, in a Civill and intestine war … to cast forth the disease’, echoed Lemnius.^45^ In these statements, disease is portrayed as an enemy, and the body as a battlefield.^46^ It may seem surprising that such metaphors were used to describe the actions of a female agent—the violence of warfare was generally deemed incompatible with femininity.^47^ However, there was one context in which female aggression was legitimate, as Garthine Walker has shown: namely, when women perceived their households to be under threat.^48^ During the Civil Wars, tales abounded of courageous wives who defended their homes, husbands, and children from enemy attack.^49^ Plays and histories valorised mothers for ‘venturing on Swords, and rushing through the flames to save their Darlings’.^50^ Since Nature was represented as the body's mother, and disease, as an adversary, it is understandable that this agent assumed the role of brave fighter during illness. Ordinary women regularly drew on these ‘saving discourses’ at court to justify their violence, a finding which indicates that the positive construction of female force was widespread.^51^ The gender paradox of the woman-warrior Nature may have also been rendered less problematic by the permeation of imagery of virtuous viragos from classical mythology and Christian scripture into early modern culture.^52^ The same could be said of the iconic status of Elizabeth I as a warrior queen.

Turning from Nature's gender to her character traits, this agent was blessed with many qualities. ‘Nature doth nothing in vain’, declared the Durham physician and minister William Bullein (*c*.1515–76).^53^ She was imaginative, ‘the best artist’, who ‘invents … certain extraordinary ingenious aid[s]’ for recovery.^54^ Nature was full of knowledge, ‘discreete, sober, and wise’, wrote the physician from Coventry, John Cotta (*c*.1575–1627).^55^ She was also caring and diligent, ‘very solicitous’ for the well-being of her hosts. Such high praise was a mark of respect to God, the ‘author of Nature’. Many of these traits were prized attributes of femininity—women were entreated to be wise, discreet, and kind.^56^ Given that Nature possessed so many qualities, it might be expected that she was an infallible agent. However, this was not the case. Nature's ability to remove disease was by no means guaranteed, but depended on her strength. The Scottish physician John Macollo (*c*.1576–1622), explained,

[T]he original of Prognosticks doth consist in conferring the spirits with the sickness; for if Nature be strong enough to overcome the disease, then the Patient shall escape; but if she be so weak that she cannot obtain the victory, death then of necessity must follow.^57^

Recovery was a tug-of-war between Nature and the disease, the outcome of which depended on strength. As Macollo implies, Nature's strength was determined by the condition of her instruments, the natural spirits, together with the innate heat. Nature was strong when the spirits were ‘many’ and ‘lively’, and the heat ‘strong’. Conversely, she was weak when the spirits were ‘dissolved and overthrown’, and the heat ‘feeble’ or ‘extinguished’.^58^

The fallibility of Nature provided the justification for the third agent in the hierarchy of healers, the practitioner. The role of this agent was expressed through the celebrated axiom, ‘the physician is nature's servant’. Derived once more from the writings of Hippocrates, this epithet is ubiquitous in medical treatises throughout the period.^59^ The physician's ‘chief office’ as Nature's servant was to ‘underprop [her] when she fails’.^60^ Nature failed when she became ‘exhausted or overwhelmed’ from her encounter with the disease. Practitioners were supposed to act as Nature's ‘faithfull friend’, ‘needfully assisting, helping, and comforting her’ against the ‘furious mercilesse’ disease.^61^ In these statements, doctors drew on popular gender stereotypes to justify their interventions, depicting themselves as chivalrous heroes who rescued ‘languishing Nature’, the damsel in distress.^62^ Since courtly love was one of the few contexts in which male subservience to females was culturally acceptable, physicians may have been invoking this language as a way to maintain their masculine identities in what might otherwise have been a demeaning situation.^63^ After all, early modern society was deeply patriarchal, and the position of ‘Nature's servant’ overturned the traditional gender order.^64^

The power balance between Nature and the physician was supposed to rest firmly with the former. A lowly servant, the practitioner was entreated to ‘act in subserviency to her Designs’, imitating Nature's methods when treating the sick.^65^ So inferior was his position, that in many cases, he was not needed at all. The Ordinary Professor of Anatomy at Utrecht, Ysbrand van Diemerbroeck (*c*.1609–74) commanded, ‘leave Nature to do her own business, in regard she does it better of her own accord then the Physitians can do by Art’.^66^ However, in practice the power balance could be reversed, with the physician taking on the dominant role. Cotta stated, ‘it is requisite in a co[m]petant Physition, that he be truly able … to be unto nature a governor … to preserve her, to conserve her, behoofefully to … guide her’.^67^ The physician was expected to restrain Nature when she became ‘exorbitant’, and ‘rouse her’ into action if she grew lazy or forgetful.^68^ This inverted relationship, which will be further explored in the next section, was encapsulated by the saying, ‘nature must play the physitian in curing of the disease’, which suggested that it was Nature who was imitating the doctor.^69^

How can this contradictory power balance be explained? Ultimately, it sprang from experience: practitioners observed that without physic, patients sometimes recovered, and sometimes died. Such instances signified that Nature's judgement was not always right, and that sometimes she needed guidance. These observations fitted with contemporary ideas about ‘the very imbecility’ of females, and their need ‘to be always directed and ordered by others’.^70^ One way to understand this ambivalent relationship is to look to the wider political context. For early modern society, the power balance between the physician and Nature may have brought to mind the case of female monarchs, most notably, Elizabeth I. Like Nature, the Queen was divinely ordained to rule, and deserved unquestioning obedience from her subjects. And yet, the inferiority and weakness of her sex warranted the intervention of her ministers, and frequently, their flagrant disobedience, especially during warfare.^71^ A more mundane, but equally powerful analogy concerns the roles of husbands and wives in the household. In his popular treatise *Of Domesticall Duties*, the London clergyman William Gouge (1578–1653), declared, ‘it is the wives … dutie to … *governe the house*’ in such matters as ‘nourishing and instructing children … adorning the house, [and] ruling maidservants’. However, he added the caveat that the husband has ‘a general oversight of all, and so [may therefore] interpose his authority’ whenever he perceives that something ‘unlawfull or unseemly … [is] done by his wife’.^72^ These two metaphors—of monarch and wife—may have acted as a model for the Nature–physician relationship.

## Processes

Having established who were the agents of recovery, it is now time to find out how they went about the task. Through exploring the physiological processes involved, it will be possible to observe in more concrete terms the complex relationship between Nature and the medical practitioner. The discussions also contribute to debates about the conceptualisation of disease, and uncover bodily mechanisms which have rarely been explored. Put simply, disease was removed by the removal of its cause. The Dean of the Faculty of Medicine of Reims, Nicholas Abraham de La Framboisière (1560–1636) confirmed, ‘while the [cause] is present, the Disease remains; but when it is remov'd, the Disease ceases’.^73^ Thus, it is first necessary to establish the cause of disease as perceived by early modern physicians. Disease was defined as a ‘condition contrary to nature which impedes function’, arising from the ‘distemperature’ of the four ‘primary qualities’ of heat, cold, moisture and dryness.^74^ All the faculties of the body and mind were thought to be driven by the special mixture of the aforementioned qualities, and therefore when this state altered, ‘perceptible impairment’ occurred.^75^ In turn, the state of the primary qualities was dictated by the quantities and conditions of a person's humours (blood, choler, melancholy and phlegm), each of which contained a different amount of heat and moisture.^76^ The notion of disease as imbalance is widely known in the historiography, but the other vital component—impairment of faculties—has often been overlooked.

Since disease was caused by the malignant alteration of the humours, it followed that the physiological mechanisms of recovery involved the rectification of these substances. Upon the command of God, Nature achieved this rectification through two main processes: the first was ‘concoction’. Concoction was defined as ‘the reduction [i.e. alteration] of the peccant humor in the body to a right temper and frame’.^77^ Whilst scholars are familiar with the use of this term in relation to the digestion of food, the concoction of humours in disease has rarely been addressed.^78^ Concoction was carried out ‘by nature it selfe, by meanes of naturall heat’, the innate warmth of living creatures—the word concoction means ‘to boil together’.^79^ Just as raw food was rendered edible by cooking, it was thought that the putrid humours would be purified by the application of heat. The depiction of concoction as cookery made sense given the persona of Nature as a housewife who attended to the nutritional needs of the body.

One of the reasons for investigating concoction is that understandings of this process shaped how the patient was managed. Concoction was thought to be an arduous and painful process, both to Nature and the patient. Hippocrates' *Aphorisms* states, ‘Whiles filthy and corrupt matter is digesting, pains and Agues do … happen’.^80^ Given the great labour involved in the process, doctors ‘command[ed] long quiet and rest to the patient’ during this phase, so that all Nature's powers could be devoted to the task.^81^ Under no circumstances should Nature be ‘diverted or hindered’ from her ‘office & work’ by such chores as eating or exercise.^82^ Practitioners warned of the hazards which would ensue if this advice was neglected. The eminent surgeon William Clowes (1543/4–1604) described how one patient, sick of ague, had ‘devoured all and every peece’ of an apple; ‘So soon as it was receyved, nature left the disease to digest the apple, which was too hard to do … at length, his Feaver … was now much worse’.^83^ Poor at multi-tasking, during illness Nature could only digest ‘thin’ foods, like broth.^84^

What was the role of physicians in concoction? Hart declared, ‘if nature be feeble[,] … and [her] heat not in a due proportion answerable, it is then the Physitians part … to supply this defect’.^85^ There were two ways to help Nature: the first was to ‘raise up’ the natural heat, so that Nature would be able to concoct the matter herself. Hart suggested that ‘in some diseases’, the passion anger ‘may be beneficial’, because it ‘stirreth up naturall heat’.^86^ The second method was to actively transform the humours, effectively doing Nature's work for her. The use of the term ‘concoction’ to denote a medicinal drink indicates most obviously this role of physic: practitioners prescribed medicines (or regimen) that exhibited the opposite qualities to the offending humours, thereby transforming them out of their malignant state.^87^ This strategy, well known to historians, was called ‘allopathic healing’. Through the above methods, practitioners were fulfilling their roles as Nature's servants, serving their mistress respectfully.

Concoction was central to prognosis as well as treatment. Doctors believed it possible to predict the outcome of disease from signs of concoction in the bodily fluids. Macollo explained,

The co[n]c[oc]tion of the humour appearing in the Excrement of the Patient, signifies the … assurance of health; but the crudity denotes that … the disease shall be longer … or finally, that death shall follow upon it.^88^

The closer the excrements resembled their state in health, the more ‘sure’ the recovery. Although in theory any of the body's excrements could be examined for this purpose, in practice it was most often the urine, because this fluid was the easiest to access. Michael Stolberg has argued that urine inspection (‘uroscopy’) was in decline from the early 1500s, as physicians sought to disassociate themselves from quacks; but I have found that it continued to be practised throughout the seventeenth century.^89^ Laypeople were also familiar with the concept of concoction, and its effects on the excrements. In 1648, the Essex clergyman Ralph Josselin (1617–83) recorded in his diary, ‘my water brake very ragged [by which] … I conceive a remainder of ill humours in mee … that nature was concocting’.^90^

We now reach the second process through which disease was overcome in Galenic perceptions: evacuation. Evacuation was defined as ‘the expulsion … of humors which are troublesome, either in quantitie, or quallitie’.^91^ It was the fate of those humours which had retained a degree of malignancy in spite of concoction, or else were simply too voluminous. Although historians are aware of the role of evacuation, its full complexity is yet to be explicated. For a start, it was believed that this mechanism should not take place until various preparative steps had occurred. Macollo stated, ‘before the body be purged, it must be prepared and the humours must be made fluxible, otherwise the purgation will not be without great pain; … wherefore … all the passages of it are to be opened, and the gross humours within are to be made liquid’.^92^ Imagery of food preparation and cookery was used in this context: Nature ‘chopped’ and ‘melted’ the thick humours, so that they could be more easily evacuated. Physicians assisted Nature by giving ‘Bitter’ medicines to devide, [and] extenuate' the ‘grosse and clammy humours’.^93^ Preparing the body for expulsion was dirty work, conjuring up images of disgusting, stinking matter being scrubbed and washed. Lemnius observed, ‘the filth and rubbish of the humours stick no lesse to … mens bodies, than the lees and dregs do to vessels, which must be soked with salt water … and rub'd … to make them clean’.^94^ This simile suited Nature's personification as a charwoman or housewife, who cooked and cleaned in the body.

Once the body was ‘fluxible’, the humours could begin their journey towards its exits. Nature drove the matter outwards, from the interior ‘noble parts’ (the most important organs, the brain, heart and liver), to the exterior ‘ignoble parts’ (the less vital regions, such as the skin).^95^ This outward motion was regarded as an ‘inherent power’ of Nature, which she used also in nutrition to remove waste products from the body.^96^ Practitioners could assist Nature by diverting the humours from the noble organs, and drawing them towards the exterior. Bruele stated that in cases of apoplexy, ‘there must … be used strong and painfull ligatures of the extreme parts, that … Nature being provoked by the vehemency of those pains, may drive out those ill humors … [from] the braine’.^97^ These interventions were supposed to be painful—it was the smart that they produced which drew the humours or cajoled Nature into action. This medical intervention is an example of the physician rising above the status of servant: he was goading Nature into doing what he thought was necessary. In family life, husbands were expected to administer ‘sharp reproofes’ to their wives as corrections for faults.^98^ Male practitioners may therefore have regarded the use of painful treatments as one of their duties as benevolent patriarchs.

At last, we have reached the point at which the humours left the body. Lemnius marvelled at the variety of exits ordained for the ejection of humours:

God that made the body of man hath not in vain created so many wayes and passages to purge forth the humours. So the head purgeth it self by the Nostrills [and] Ears[;] the Pa|late, [by] … [s]neesing and spitting; The … Lungs by … coughing; the Stomach … by vomit … ; The Intestines … by the belly … ; The Reins … by the urinary passages … ; all … sweat through the skin that is full of holes.^99^

Expulsion was carried out by Nature during what was known as the ‘crisis of disease’ or ‘critical expulsion’. The crisis was the ‘swift and suddain’ evacuation of humours at the height of illness, in the form of sweating, urination, vomiting, diarrhoea, haemorrhage, the bursting of pustules or, in the case of women of reproductive age, the sudden onset of menstruation.^100^ The crisis was regarded as the turning point in illness, ‘whereby the sick is either brought to recovery, or death’.^101^ Unlike concoction, this mechanism was described in masculine language, commonly linked to combat: the ‘healthfull crisis’, wrote Cotta, is ‘the victorie of nature in the masterie of her enemie the disease’.^102^ The crisis was depicted as the decisive battle between Nature and the disease, upon which the outcome of illness depended; such imagery was appropriate given the personification of this agent as a warrior. Sabine Kalff has shown that the theory of the crisis was so convincing that it was appropriated by political thinkers to justify military decisions.^103^

Descriptions of the crisis shed light on how disease was conceptualised. Although in principle the evacuated humour was the cause of disease rather than the disease itself, authors often implied that it could be both. Culpeper, for instance, states, ‘nature labours to expell the humour that causeth the disease’, but in another place, writes, ‘nature did the best she could to expell the disease’.^104^ By implying that the humours constituted the material basis of illness, such statements challenge the widespread notion that disease was conceived wholly non-ontologically in Galenic thinking.^105^ This research thus supports Michael Stolberg's view that disease was thought of, at least in part, as an entity in the early modern period.^106^

What is most striking about the theory of the crisis is that it suggested that evacuative diseases cured themselves. ‘Vomiting [is] cured with vomiting, and purging with purging’, declared the royal physician Tobias Whitaker (1601–64).^107^ It is probable that this notion brought comfort to patients: they could rest assured that however unpleasant the crisis, it would lead to their recovery. Doctors and laypeople waited for the crisis, and longed for it to occur. The country gentleman, James Clavering (1680–1748), received a letter from his brother-in-law in 1725 about the condition of his young son James, who was suffering from a lung illness: he said, ‘I wish this humour wou[l]d grow and swell more to a head and then burst, as there is no hopes left without an extraordinary evacuation somewhere’.^108^ As well as demonstrating the endurance of ideas about the crisis into the eighteenth century, this example suggests that without a critical expulsion, recovery was reckoned to be impossible.

The theory of the therapeutic crisis had implications for medical treatment. Namely, it was considered inadvisable to relieve the evacuative symptoms of illness.^109^ Speaking of diarrhoea, the medical writer from Suffolk, Phillip Barrough (d.1600), insisted, ‘you must suffer and watch, till nature hath bestowed all her care’; he warned, ‘to stoppe the fluxe, it causeth a worse and greater disease’.^110^ This idea was expressed by laypeople as well as physicians.^111^ It was feared that if the evacuations were terminated prematurely, the humours would be ‘turned backe’ into the interior, where they would do damage to the noble organs.^112^ By allowing Nature to evacuate the matter unhindered, the physician was adhering to his role of deferential servant. In the history of medicine, it is sometimes suggested that patients who were suffering from an evacuative symptom were prescribed treatments designed to promote that same evacuation.^113^ This was not usually the case. In theory, practitioners only intervened to instigate an evacuation when Nature was struggling to produce that emission.^114^ Van Diemerbroek reported that one Monseiur de Guade, a French captain, was taken with nausea and a great desire to vomit, but ‘he [could] not Vomit up very much’; the doctor administered a ‘good draught of the Decoction of Barley’, and the result was that he was able to vomit ‘a great quantity’.^115^ The patient's nausea was a sign that Nature wanted the patient to vomit, and indicated that the practitioner should assist by administering an emetic. On the other hand, if the patient had already been vomiting profusely, there was no need to promote the evacuation because Nature was coping on her own.^116^ This subtle distinction is important because it has implications for the patient's experience of medical intervention: if practitioners only prescribed those evacuative treatments which their patients ‘desired’, early modern medicine begins to appear less barbaric than it has been conventionally portrayed.^117^

On those occasions when it *was* necessary to administer an evacuative treatment, practitioners were instructed to consider the ‘inclinations of Nature’, which meant choosing the type of evacuation that this agent seemed to be attempting.^118^ This rule, which exemplifies the subservient position of physicians, endured into the eighteenth century.^119^ How could practitioners discern Nature's inclinations? Thinking back to Anthony Walker, he had demanded to be blood-let on the grounds that he had suffered a nosebleed, a sign that Nature wished to purge his body of blood.^120^ Each evacuation was signalled by a particular sign—sweating, for example, was suggested through ‘moystnesse of the skin’, while diarrhoea was hinted by ‘gripings … [and] the murmuring … of the guts’.^121^ Doctors warned that to fail to follow Nature's intentions would ‘move or irritate’ this agent so that she would ‘greedily keep back’ the humours, and refuse to cooperate with the practitioner.^122^ In such statements, physicians draw on imagery of the recalcitrant female to convey their displeasure at Nature's response, even though it is actually they themselves who are to blame, since they had broken the rule of following this agent's inclinations.

There was an important caveat to the rule of following Nature's inclinations, which provided practitioners with an opportunity to rise above the status of servant: namely, that her inclinations were not always trustworthy. Sprengell commented, ‘she … *Errs* now and then in selecting *improper Organs,* and attempting her … *Excretions* through incongruent … Passages’.^123^ Typical examples included the use of ‘the Nipples, the Mouth, or the Eyes’ for the voiding of blood. In these situations, practitioners sought ‘to oblige *Nature* to alter Her Purpose’.^124^ The early modern body is often depicted as a hollow container, within which fluids could move around freely and unconstrained.^125^ The above discussions indicate that this reading is an oversimplification, since it is clear that doctors distinguished between the different passages of the body, and considered that some were more appropriate than others for the evacuation of humours.

So far, it has been implied that recovery always involved evacuation. However, this was not the case—sometimes it required the opposite process: retention. Hippocrates' *Aphorismes* states, ‘Diseases which are bred of satiety … are cured by evacuation and those which proceed from emptiness are cured by fulness.’^126^ Retention meant the termination of evacuative symptoms, and the replenishment of lost humours. It was required when the crisis had been too violent, or when the disease itself was caused by a shortage of humours.^127^ Nature was to blame—in keeping with her female identity, she was inclined to be ‘exorbitant’ with her evacuations, removing too great a quantity of the humours. Speaking of Galenic notions of Nature, Boyle observed, ‘Physicians oftentimes … employ their best Skill … to suppress … the inordinate Motions … that … *Nature* rashly begins to make’. So far ‘from taking *Nature* for his Mistress’, the physician spends ‘a great Part’ of his time ‘hinder[ing] her from doing what She seems to Design’, he wrote.^128^ Thus, once again, the power balance was overturned.

The process of retention was the reverse of the process of evacuation: the humours had to be made *in*fluxible. Barrough suggested that this could be achieved ‘by cooling [the humour], or by thickening it, or else … by shutting & occluding the … wayes wherby it would flow out’.^129^ Van Diemerbrock recorded that when the French army was taken with dysentery, the physicians ‘took white Wax … cut … very small’, and boiled it in milk ‘till the Wax was perfectly melted’, and then ‘gave their Patient that Milk … hot … to drink’. The idea was that as the wax cooled in the body, it would set, thereby thickening the humours.^130^ Physical barriers could be placed at the exit points, such as ‘Tents of new Cloth’ put up the nostrils to staunch nosebleeds.^131^ Violent evacuations could also be terminated by inducing alternative ones. A typical example was to administer a laxative to a patient who was vomiting—the humours would be diverted from the stomach to the bowels, where they could be ‘turne[d] out … at the back-door’.^132^

Laypeople as well as practitioners attempted retention, thus indicating once again the shared understanding of recovery between these groups. Domestic recipe books are replete with remedies for the ‘staunching of fluxes’.^133^ Letters provide more poignant insights into the experiences of families as they strived, and sometimes failed, to restrain Nature's evacuations. In 1647, the Buckinghamshire politician Ralph Verney (1613–96), wrote to his uncle about the severe diarrhoea of his eight-year-old daughter Pegg: he explained that despite giving ‘Asses Milke’, a well-known thickening agent, ‘wee can by noe means stay it, nor thicken the Humours, [for] … she comonly goes to stoole 16, 18, or 20 times in 24 howors’. He concluded his letter with the mournful words, ‘I am soe full of affliction that I can say no more but pray for us’.^134^ Although it is harder to uncover the practices of lower socioeconomic groups, second-hand evidence suggests they too sought to end violent evacuations. The Huntingdon physician John Symcotts (*c*.1592–1662) recorded in his casebook that in 1639, a certain ‘cook-maid’ staunched the nosebleed of the 13-year-old daughter of her Mistress, Lady Cotton. The maid ‘boldly took a cloth wet in cold water’, and made the girl ‘sit upon it, and so [it] was stayed’.^135^ It was believed that there was a connection between the nose and the womb; since the girl was at the age of menarche, it is possible that the maid was trying to divert the blood from the nose to the womb through this treatment, where it could be safely evacuated through menstruation.

## Helmontians

To demonstrate the pervasiveness of the tripartite model of the agents of recovery, this final section examines the beliefs of a group of physicians who rejected many of the other key features of Galenic medicine, the Helmontians. Helmontians were supporters of the Flemish doctor and chemist Jan Baptista van Helmont, who in turn had been strongly influenced by the Swiss medical reformer Paracelsus (1493–1541). This movement was part of the development of the ‘new science’ which repudiated the learning of the ancients. It was also deeply religious: Helmont believed that Galenism's roots were pagan, and he was convinced he had been sent by God to bring a truly Christian form of medicine into the world. In England, the influence of Helmontianism peaked in the 1660s, when 35 physicians nearly succeeded in establishing a Society of Chemical Physicians.^136^ Ultimately, however, this brand of medicine failed to topple Galenism, and by the close of the seventeenth century, it had been largely absorbed into humoral medical practice.^137^ Amongst the laity, I have found little evidence to suggest that Helmontian medicine was ever widely adopted—patients' diaries and letters discuss recovery within a humoral, rather than a Helmontian, framework.^138^ Through comparing Galenic and Helmontian notions of recovery, it will be possible to arrive at a better understanding of why the agency of Nature proved so resilient, whilst highlighting possible reasons for the Helmontian failure.

Helmontians agreed with Galenists about the roles of God, Nature, and the physician in recovery. Helmont affirmed ‘that thing Hippocrates so long agoe smelt out … that Nature alone … is the Physitianess of Diseases, but the Physitian the Minister or Servant’.^139^ There were, however, differences in vocabulary: Helmontians usually substituted the term ‘Nature’ with the word ‘Archeus’, a term defined as ‘the Arch Preeminent Author of Health’.^140^ Like Nature, the Archeus carried out all the body's basic functions, including digestion, reproduction and recovery. Helmontians used the terms ‘Nature’ and ‘Archeus’ interchangeably, and on occasions made it clear that the two were synonymous. The chemical practitioner Thomas Cock (b*.* 1630), wrote in his popular chemical treatise, ‘Nature, i.e. [the] *Archeus*’, and in another place referred to the ‘*Archeus* or Nature’.^141^ Likewise, in dictionaries these two concepts are merged.^142^ Nevertheless, the Archeus and Nature were distinguished in their sex—the former was male, although, as we will see below, he was described using feminine imagery. A possible reason for favouring the male Archeus over the female Nature was its lack of pagan connotations.^143^

While agreeing about the roles of Nature and the physician, Helmontians and Galenists diverged in relation to the causes of disease. Helmont asserted, ‘a Disease is not a certain distemperature of elementary qualities, … as hitherto the *Galenists* have dreamed’, but rather, ‘it is a strange Image … out of the Archeus’.^144^ Dismissing the humours as ‘frivolous fictions’, human bodies were instead composed of just two elements: water and ‘Ferment’, an ‘active, brisk, aetherial substance’.^145^ Helmont taught that after the Fall of mankind, the Archeus had been ‘pierced or defiled’ by innumerable ‘diseasie ideas’.^146^ Compared to the seeds of plants, these immaterial ideas were like blueprints for every sort of disease. In health, they lay dormant, but as soon as the Archeus began to think about them, they ‘hatched’, ‘spread[ing] into various Branches, and Fruits’, and harming the organs.^147^ The Archeus began to dwell on these ideas when it became ‘sorrowful, angry, … [or] vexed’.^148^ Helmontians portrayed this agent as an enraged woman who had become ‘violent and disobedient’. Such ideas were informed by the notion that females had particularly powerful imaginations, and were prone to anger.^149^ Helmont's deep religiosity, and his belief in the sinful state of mankind, also shaped this theory. In sum, whereas Galenists regarded disease as a state of malfunctioning caused by the alteration of the humours, Helmontians defined it as a ‘strange idea’ fashioned by the Archeus or Nature herself.^150^

In the light of these different ideas about disease causation, it followed that Galenists and Helmontians held different views about how recovery occurred. As we saw earlier, for Galenists, the processes involved rectifying the humours. By contrast, Helmontians taught that illness was overcome by removing ‘the … Idea of the Disease in the *Archeus*’.^151^ There were two ways to do this. The first was to strengthen the Archeus, so that it was better able to resist the disease ideas. Helmontians used ‘sympathetic’ remedies: the opposite to Galenic allopathic treatments, these were medicines which ‘have Similtude’ with the Archeus, such as highly purified minerals. It was believed that, ‘seeing Like doth readily unite with Like’, the Archeus and medicine would ‘embrace each other intimately’, whereby the ‘Spirits’, the instruments through which the Archeus worked, ‘in a moment [are] encreased’ and strengthened.^152^ By depicting the Archeus and medicine as friends, Helmontians hoped to make their remedies appealing to their patients.

The second method for removing disease was to ‘pacify and gratify’ the Archeus, so that it ‘layeth aside’ its rage.^153^ This was achieved through giving ‘exquisite’, ‘delectable’ medicines, of ‘grateful smell and taste’.^154^ The leading English Helmontian, George Thomson (1619–77), imagined that the medicine would be ‘Conducted into the very Bed-Chamber’ of the body, where its beauty would be shown in a ‘Looking-Glass’ to the Archeus; the contrast between ‘the ugly shape of the Disease’, and the beautiful medicine would be so striking that the Archeus would ‘Repent of Former Errors’, and end its diseasie thoughts.^155^ Rather than using language of warfare and housework, Helmontians deployed imagery of light, beauty and feasting. Medicines were ‘beautiful objects’ that ‘send forth Lively Illustrious Beams’ into the body, ‘scatter[ing] those black clouds of mischievous Idea's’.^156^ Physic was a ‘Dainty Morsel for the Archeus to Banquet on … feasting upon [it] with admirable delight’.^157^ Light was the emblem of Helmontianism, chosen for its religious associations—Christ ‘is the truth, the life, the light’, who had enlightened Helmontians to understand the true art of curing.^158^ The references to beauty tap into popular gender stereotypes, namely the vanity of women, and their penchant for pretty things like jewellery.^159^ In these descriptions, the personalities of the Galenic Nature and the Archeus seem very different: the antithesis to the hardworking cleaner, the Archeus was a spoiled queen. Given that Helmontian physic sounds far more pleasant than Galenic medicine, it might seem incongruous that the latter remained more popular. Andrew Wear suggests that people were so accustomed to the notion that the ‘medicine must be as bitter as the disease’, that to take a mild or pleasant medicine would instantly have raised doubts about its efficacy.^160^

Why did Helmontians retain the precept that ‘Nature is the healer of the disease’, whilst rejecting other fundamentals of Galenism? One important reason was that it offered valuable opportunities for anti-Galenic propaganda. Helmontians sought to undermine the very foundation upon which Galenic physic rested by accusing its practitioners of failing to fulfil their roles as Nature's servants. ‘Far from assisting … Nature’, the Galenic doctor ‘becomes a *hindrance* … to her’, declared Thomson.^161^ Helmontian attacks centred on the effects of evacuative treatments, which they believed were ‘pernicious to Humane Nature, destroying more then ever the Sword’.^162^ Ultimately, Galenic intervention exacerbated the cause of illness: it ‘enrage[s] the Archeus, stirring up Storms … in the Microcosm’, thereby encouraging this agent to dwell even more on the ‘diseasie ideas’.^163^ Rhetorically, it was useful that Nature was personified—it made for a more emotive argument: this fragile female was ‘fretted, gall'd, or opprest with … *disgusting* medicines’; she was ‘betrayed’, ‘worried by a Disease, and thrown flat on [her] … back … by cruel Phlebotomy, [and] poisonous Purgations’.^164^ In short, the agency of Nature was preserved by Helmontians because it was simply too useful to give up—they could fight Galenists at their own game by trying to undermine their mission as physicians.

## Conclusion

The ‘golden Saying’ in early modern medicine was ‘Nature is the healer of disease’.^165^ By drawing attention to this forgotten axiom, I have sought to enrich our understanding of the rationale behind early modern medicine. Medical treatments were supposed to mimic the actions of Nature, concocting and expelling the bad humours. This enhanced understanding will help us to overcome a pressing challenge faced in the history of early modern medicine: the urge to cast judgements on past medical practices. Whilst it is no longer acceptable in academic circles to ridicule early modern medicine, in the sphere of popular history, this attitude continues to dominate. By properly appreciating the rationale behind treatment, those medicines which might have initially appeared ludicrous—such as giving a laxative to a patient who is vomiting—are rendered more understandable. The significance of these findings extends beyond medical history, to the history of physiology more generally. Nature was responsible for carrying out all the basic bodily functions. Indeed, the importance of this agent in physiology is indicated most obviously by the fact that the word ‘physis’ means ‘nature’ in Latin. By properly appreciating the meaning of the word ‘nature’, early modern descriptions of physiological processes begin to make more sense. Nature's role also has implications for religious history. For instance, it will provide deeper insights into the meaning of ‘supernatural’ cures. If we study what Nature *could* accomplish, we will be in a better position to understand events which were classed as ‘above’ this agent. This article has concentrated mainly on the relationship between Nature and medical intervention; it invites further studies on the interactions between Nature and God.

A theme running through the article has been the complex gender and power dynamics between Nature and the physician. In theory, the physician was ‘Nature's servant’; but in practice the power dynamics were often more ambivalent, with the physician frequently appearing more like Nature's co-governor, or even the superior party. These complexities reflect wider cultural paradoxes surrounding womankind: female Nature was benevolent and caring, but also, impetuous and weak. This fallibility legitimised the interventions of the practitioner, and may also have provided opportunities for gender construction: the doctor could see himself as a gallant hero who rescued the swooning Nature, or as a wise patriarch who restrained her ‘outrageous’ acts. In a sense, it was entirely appropriate that Nature was female: concoction was akin to cooking, and expulsion resembled washing. However, we have seen that recovery was also described in masculine language—it was a battle between a princely Nature and the enemy disease. Contemporaries may have reconciled this gender paradox by considering that Nature, like a loving mother, would fight to the death to protect her child, the human body. Through these discussions, the article suggests that medical theory is a lens through which we can glimpse wider ideas about gender. Medical beliefs mirrored, and possibly also, reinforced, gender stereotypes: doctors and laypeople witnessed on a daily basis the benevolence, and fallibility of female Nature, and such observations may have informed how they thought of women. I hope the study will also spark new questions in the histories of gender and sex difference. For example, if males as well as females were believed to be inhabited by a female Nature, how might this have affected their gender identities? Was the physiological distinctiveness of the two sexes lessened by the belief that both were governed by a female Nature? The discussions have focused on the relationship between male practitioners and Nature. It would be fruitful to explore the interactions between this agent and female practitioners, perhaps asking whether the latter felt a special affinity with Nature as a fellow female healer.

The removal of disease was ‘busied about the humours’—the noxious matter was corrected through processes of concoction and expulsion or retention. Historians are more familiar with expulsion than with the other two processes, but I hope to have shown that expulsion was rather more complicated than has been recognised. People pictured the ‘thicke grosse’ humours being chopped and scrubbed by Nature, and pushed outwards from one organ to the next, until at last they arrived at the bodily exits. These lucid imaginings demonstrate the importance of the organs and vessels in early modern concepts of the body. Such findings support the recent ‘body in parts’ approach to the history of medicine which challenges the entrenched notion that the organs and vessels hardly featured in early modern concepts of the body.^166^ The research has also contributed to debates about concepts of disease, concurring with Michael Stolberg that illness could be envisaged ontologically in the early modern period.

Finally, this article has compared Helmontian and Galenic ideas about recovery as a way to demonstrate how deeply ingrained the notion of Nature had become in early modern medicine. Helmontians rejected some of the core components of Galenic medicine, and yet they did not part from the precept that ‘Nature is the healer of disease, and the physician but the servant’. This may have been because the axiom was flexible, and could be applied to any medical theory. A more important reason, however, was that the personification of Nature served as powerful propaganda upon which rival groups could promote themselves and denigrate their opponents. Helmontians accused Galenists of being cruel oppressors of ‘sweet Nature’, and depicted themselves as her ‘kindly friends’.^167^ In view of the competitive character of the medical marketplace of early modern England, there was a great demand for this sort of emotive strategy.^168^ Given that Helmontian medicine was probably more pleasant than Galenic physic, we might wonder why the latter continued to dominate in this period. One reason could be that the Helmontian theory was emotionally less palatable than the Galenic one. Although Galenists also believed that humankind was responsible for disease—God brought illness as a punishment for sin—their version of Nature was not so explicitly culpable. Nature cured, rather than caused, disease. By contrast, the Helmontian Archeus appeared a sinister figure, who could at any moment bring disease simply by thinking about it. Ultimately, the appeal of Galenic theory lay in its capacity to make illness seem less terrifying to the sick: it transformed the most painful part of illness—the crisis—into the method of cure.
